# Repetitive Exposure to Orofacial Somatosensory Inputs in Speech Perceptual Training Modulates Vowel Categorization in Speech Perception

**DOI:** 10.3389/fpsyg.2022.839087

**Published:** 2022-04-26

**Authors:** Takayuki Ito, Rintaro Ogane

**Affiliations:** ^1^Univ. Grenoble Alpes, CNRS, Grenoble INP, GIPSA-lab, Grenoble, France; ^2^Haskins Laboratories, New Haven, CT, United States

**Keywords:** somatosensory stimulation, perceptual adaptation, multisensory integration, production-perception link, auditory representation

## Abstract

Orofacial somatosensory inputs may play a role in the link between speech perception and production. Given the fact that speech motor learning, which involves paired auditory and somatosensory inputs, results in changes to speech perceptual representations, somatosensory inputs may also be involved in learning or adaptive processes of speech perception. Here we show that repetitive pairing of somatosensory inputs and sounds, such as occurs during speech production and motor learning, can also induce a change of speech perception. We examined whether the category boundary between /ε/ and /a/ was changed as a result of perceptual training with orofacial somatosensory inputs. The experiment consisted of three phases: Baseline, Training, and Aftereffect. In all phases, a vowel identification test was used to identify the perceptual boundary between /ε/ and /a/. In the Baseline and the Aftereffect phase, an adaptive method based on the maximum-likelihood procedure was applied to detect the category boundary using a small number of trials. In the Training phase, we used the method of constant stimuli in order to expose participants to stimulus variants which covered the range between /ε/ and /a/ evenly. In this phase, to mimic the sensory input that accompanies speech production and learning in an experimental group, somatosensory stimulation was applied in the upward direction when the stimulus sound was presented. A control group (CTL) followed the same training procedure in the absence of somatosensory stimulation. When we compared category boundaries prior to and following paired auditory-somatosensory training, the boundary for participants in the experimental group reliably changed in the direction of /ε/, indicating that the participants perceived /a/ more than /ε/ as a consequence of training. In contrast, the CTL did not show any change. Although a limited number of participants were tested, the perceptual shift was reduced and almost eliminated 1 week later. Our data suggest that repetitive exposure of somatosensory inputs in a task that simulates the sensory pairing which occurs during speech production, changes perceptual system and supports the idea that somatosensory inputs play a role in speech perceptual adaptation, probably contributing to the formation of sound representations for speech perception.

## Introduction

Speech perception is auditory in nature but it is also an interactive process involving other sensory inputs. For example, visual information coming from a speaker’s face helps in the identification of speech sounds in a noisy environment (Sumby and Pollack, 1954). Incongruent visual information from facial movements likewise affects speech perception (McGurk and MacDonald, 1976). Recent studies have demonstrated that somatosensory inputs also contribute to the perception of speech. When air-puffs, similar to those associated with a plosive speech sound (such as /p/), were presented to the skin, perception was biased in the direction of the corresponding sound (Gick and Derrick, 2009). When somatosensory stimulation using facial skin deformation was applied in conjunction with the speech sounds, vowel perception was systematically biased (Ito et al., 2009). In a vowel identification task on a “head/had” continuum, the presented vowels were perceived more as “head” when an upward skin stretch was applied, more as “had” when the skin stretch was downward, and there was no effect with backward skin stretch. A similar effect has been observed in both children and adults using a vowel continuum between /e/ and /ø/ (Trudeau-Fisette et al., 2019). When the skin stretch was backward, the presented sounds were perceived more as /e/, a vowel in which lip spreading is involved in production. A somatosensory influence on perception is not limited to vowel categorization, but is also observed in word segmentation in lexical processing (Ogane et al., 2020). The segmentation boundary changed depending on the placement of somatosensory stimulation in relation to the key vowel in a test phrase. While these studies suggest a potential role of somatosensory inputs in speech perception, the specific contribution of the somatosensory system is unknown.

Given that orofacial somatosensory inputs normally provide articulatory information in the context of speech production (Johansson et al., 1988; Ito and Gomi, 2007; Ito and Ostry, 2010), somatosensory effects on speech perception may be production related. This idea was initially proposed in the Motor Theory of Speech Perception (Liberman et al., 1967), and extended in the Direct Realist perspective (Fowler, 1986) and the Perception-for-Action-Control theory (Schwartz et al., 2012). The possible contribution of the sensorimotor system to perception has mostly focused on the motor system. For example, activity in brain motor areas has been observed during speech perception (Wilson et al., 2004; Skipper et al., 2005), and the perception of speech sounds can be modulated by applying transcranial magnetic stimulation to the premotor cortex (Meister et al., 2007; D’Ausilio et al., 2009). At a behavioral level, when speech articulation is simultaneous with listening, the perception of speech sounds is altered (Sams et al., 2005; Mochida et al., 2013; Sato et al., 2013). However, speech motor outflow always occurs in conjunction with correlated somatic input. While somatosensory function might be considered part of motor system, the somatosensory system may work independently in the perception of speech sounds since there is a direct influence and interaction between the somatosensory and auditory system in situations other than speech perception (Foxe et al., 2000; Beauchamp et al., 2008). Thus, investigating somatosensory function in speech perception may be important in clarifying the link between speech production and perception.

The contribution of somatosensory inputs to speech perception has been examined in the context of motor learning. Previous studies showed that adapting to different external environments during production changes the vowel category boundary (Nasir and Ostry, 2009). Similar perceptual changes have been reported in studies of adaptation to altered auditory feedback (Shiller et al., 2009; Lametti et al., 2014). Although both motor outputs and somatosensory inputs are involved in the speech motor learning tasks used in these previous studies, Ohashi and Ito (2019) specifically demonstrated that somatosensory inputs on their own can contribute to the recalibration of perception. That study applied additional somatosensory stimulation during adaptation to altered auditory feedback and assessed changes to the category boundary of fricative consonants. They observed perceptual recalibration in conjunction with somatosensory stimulation, suggesting that repetitive exposure to somatosensory inputs during learning can be a key to changing or recalibration of the speech perceptual representation.

In addition to motor learning, repetitive exposure to sensory stimuli also induces changes to sensory processing. In the case of speech, the phonetic boundary between two neighboring speech sounds can be biased away from the one that is repetitively presented as an adapter in training, which is known as selective adaptation (Eimas and Corbit, 1973). Similar effects can be seen in visual speech perception (Jones et al., 2010). This type of sensory adaptation has been frequently investigated in non-linguistic processing. In the visual domain, after looking at a high-contrast visual image, a low-contrast portion of a test image briefly appears invisible (e.g., Kohn, 2007). Similarly, after prolonged observation of a waterfall, an illusory upward motion can be induced when observing a static image (Mather et al., 1998). This effect has also been demonstrated in multisensory environments, including selective adaptation in audio-visual speech (Roberts and Summerfield, 1981; Saldaña and Rosenblum, 1994; Dias et al., 2016). In case of ambiguous speech sounds, visual information from speech movements changes auditory perception (Bertelson et al., 2003). Speech sounds also change visual speech perception (Baart and Vroomen, 2010). If somatosensory inputs contribute to the formulation or calibration of speech perceptual representations, repetitive exposure to orofacial somatosensory stimulation, such as occurs normally in conjunction with speech production and learning, may recalibrate the representation of speech sounds. If this adaptive change persists following perceptual training, then training with somatosensory stimulation may potentially be used as a tool for speech training and rehabilitation.

The present study examined whether repetitive exposure to orofacial somatosensory stimulation during a speech perception task changes the perception of speech sounds. To test this idea, we here focused on the category boundary between the vowels /ε/ and /a/ and applied orofacial somatosensory stimulation, specifically facial skin deformation, as used in previous studies (Ito et al., 2009; Trudeau-Fisette et al., 2019; Ogane et al., 2020). The use of orofacial somatosensory stimulation is premised on the assumption that skin receptors provide kinesthetic information (Johansson et al., 1988; Ito and Gomi, 2007; Ito and Ostry, 2010). Given that somatosensory stimulation involving facial skin deformation changed the category boundary between “head” and “had” in on-line manner (Ito et al., 2009), training with the same auditory-somatosensory pairing may change or recalibrate the vowel category boundary in purely auditory perceptual tests. We carried out perceptual training paired with somatosensory stimulation and assessed changes to the category boundary. It might be expected, based on our prior work using a simple perceptual classification task (Ito et al., 2009), that upward skin stretch during vowel identification on a /ε/ to /a/ continuum would bias perception toward /ε/. However, if the effect of the training task on perception is similar to selective adaptation mentioned above, training might be accompanied by a perceptual shift toward /a/. Either perceptual change would suggest that the somatosensory system contributes to the link between speech production and perception, and that somatosensory inputs can help in the processing of speech sounds in ambiguous situations.

## Materials and Methods

### Participants

Thirty native speakers of French participated in the experiment. The participants were all healthy young adults who reported normal hearing. All participants signed informed consent forms approved by the Local Ethical Committee of the University Grenoble Alpes (CERNI: Comité d’Ethique pour les Recherches non Interventionnelles: Avis-2015-03-03-62 or CERGA: Comité d’Ethique pour la Recherche, Grenoble Alpes: Avis-2018-12-11-4).

### Auditory Stimulation

We focused on vowel categorization using an /ε/ to /a/ continuum, based on a previous study which showed a clear somatosensory effect on speech perception (Ito et al., 2009). These vowels were followed by the /f/ sound which is associated with a closing movement after the vowel production. The stimulus continuum was synthesized by using an iterative Burg algorithm for estimating spectral parameters. The procedure involved shifting the first (F1) and the second (F2) formant frequencies in equal steps from values observed for /ε/ to those associated with /a/. The stimulus sound was recorded by male speaker of French. The first and second formant values for the endpoint stimuli were 561 Hz and 1630 Hz for /ε/, and 712 Hz and 1203 Hz for /a/. A forty-six-step continuum was produced for the adaptive testing procedure used in Baseline and Aftereffect tests; a subset of these stimuli was selected for use in perceptual training (see below).

### Somatosensory Stimulation

**FIGURE 1:**
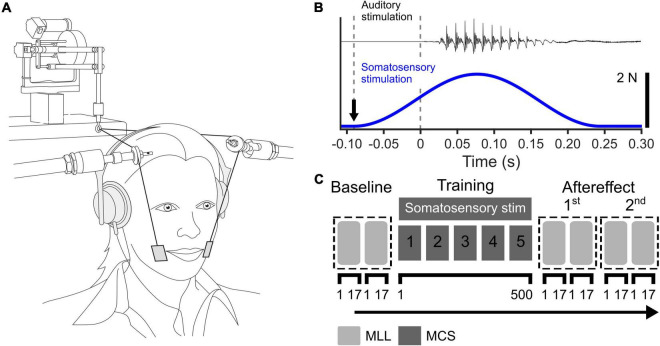
**(A)** Experimental setup for somatosensory stimulation using facial skin deformation, reproduced from Ito et al. (2009). **(B)** Time course of auditory stimulus (top) and applied force during somatosensory stimulation (bottom). The black arrow represents the onset of somatosensory stimulation. **(C)** Experimental procedure in the auditory-somatosensory perceptual adaptation test. MLL represents the maximum likelihood procedure and MCS represents the perceptual test based on the method of constant stimuli.

We used facial skin stretch applied by a robotic device to produce somatosensory stimulation (Phantom 1.0; SensAble Technologies). The experimental setup is shown in Figure 1A. Plastic tabs (2 cm × 3 cm) were attached to the skin lateral to the oral angle on each side of the face. These tabs were connected to the robotic device through thin wires. The wires were supported by wire supports to avoid contact with the facial skin. The skin was stretched when the robotic device applied force to the wires. The temporal profile of the applied force was a single cycle of a 3-Hz sinusoid with 2N peak force (see Figure 1B). Based on the previous finding that the upward skin stretch induced a relatively large change in vowel categorization judgments between “head” and “had” (Ito et al., 2009), we applied the skin stretch in an upward direction.

### Perceptual Test and Adaptation Training

The main test was consisted of three phases: Baseline, Training, and Aftereffect (see Figure 1C). In all three phases, an identification test using the vowels /ε/ and /a/ was involved. The stimuli were presented through head-phones at a comfortable volume. On each trial, participants were asked to identify whether the sound was /εf/ or /af/ by pressing a key on a keyboard.

In the main perceptual training portion of the study, the method of constant stimuli (MCS) was used in order to expose participants to values between /ε/ and /a/ evenly during the training. We used 10 of 46 steps on the /ε/ to /a/ continuum (Nos. 1, 6, 11, 16, 21, 26, 31, 36, 41, and 46) and presented them 10 times each in pseudo-random order. Each training block consisted of 100 trials. This was repeated 5 times. In total, 500 stimuli were presented. For the experimental group which received somatosensory training (SOMA), somatosensory stimulation was applied on each trial. The temporal relationship between the sound stimulus and somatosensory stimulation is shown in Figure 1B. For the control group (CTL), we carried out the same training including the setup of the robot, but in absence of somatosensory stimulation.

In Baseline and Aftereffect tests, we used an adaptive method based on the maximum-likelihood (MLL) procedure to estimate the vowel category boundary (Shen and Richards, 2012). The benefit of this procedure is its ability to estimate the psychometric function and the associated category boundary with a relatively small number of responses in comparison to other conventional methods such as MCS. However, sounds near to the perceptual boundary are primarily tested. In this procedure, the auditory test stimulus on each trial is determined in an adaptive fashion based on the stimulus that provides the most information about the shape of the psychometric function. All stimuli on the forty-six-step continuum were used in this procedure. Each of the perceptual tests consisted of four 17-trial blocks. The first two blocks of the Baseline phase were removed from the analysis as familiarization trials for the identification task.

In order to examine if the effect of paired auditory-somatosensory training persisted 1 week later, we also repeated the Post-test using the same procedure as in the Aftereffect phase, based on MLL procedure. Five of 15 participants participated in the Post-test.

### Data Analysis

We calculated the probability that the participant identified the presented vowel as /a/. We estimated the psychometric function for each 17-trial block of the MLL procedure (Baseline, Aftereffect and Post-test) and for each 100-trial block of the MCS procedure (Training), and obtained estimates of the category boundary as the 50% value of the psychometric function. The baseline value for the category boundary was obtained by averaging the two blocks of the Baseline phase. In the Aftereffect and Post-test phases, we also averaged separately the first two (1st set) and the second two blocks (2nd set). The obtained category boundaries were normalized by dividing by the baseline boundary value.

To examine whether the category boundary changed following perceptual training, we applied a one sample *t*-test to the normalized perceptual boundary immediately following training (average of the first two blocks of the Aftereffect). This normalized perceptual boundary was also compared between control and somatosensory training groups using a Linear Mixed-Effects (LME) Models analysis with nlme package in R (Pinheiro et al., 2022). In the LME model including the following analyses, participants were always considered as a random effect.

We also applied a LME analysis to evaluate whether the perceptual boundary changed over the course of training (Training phase). Fixed factors were groups (CTL and SOMA) and blocks (1, 2, 3, 4, and 5). A separate one-sample *t*-test was also used to examine whether the category boundary averaged over the course of training was different than baseline.

The LME analysis was likewise used to evaluate the possible presence of persistent effect at a one-week delay. For this evaluation, we first compared changes between the 1st and 2nd sets in the Aftereffect phase, with groups (CTL and SOMA) and sets (1st and 2nd) as fixed factors. *Post hoc* tests with Bonferroni correction were carried out to compare all possible combinations using the multcomp package in R (Hothorn et al., 2008). Second, we compared the Aftereffect and Post-test phases in the five participants that completed both. In this analysis, we extracted the category boundaries for these participants in the Baseline, Aftereffect and Post-test measures and calculated separately the normalized boundary in the Aftereffect and Post-test, as described above. We used a LME analysis to assess whether the normalized boundary was different in the Aftereffect and Post-test measures. Fixed factors in this analysis were phases (Aftereffect and Post-test) and sets (1st and 2nd).

## Results

### Shift of Category Boundary Due to the Training

**FIGURE 2:**
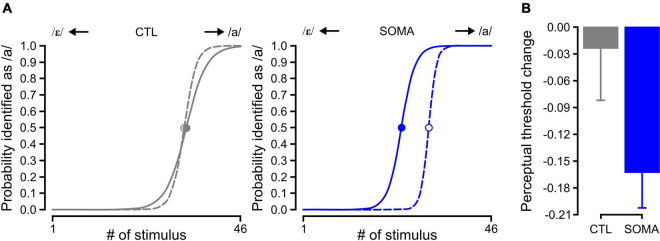
**(A)** The estimated psychometric function in Baseline (dashed) and Aftereffect (solid) phases for control and somatosensory conditions in representative participants. Filled (Aftereffect) and open (Baseline) circles represent the 50% crossover value of the psychometric function. The left panel in gray shows the participant response in the control condition (CTL); the right panel in blue shows the response in the condition that received somatosensory stimulation (SOMA). **(B)** Averaged perceptual change of the 50% crossover values for the control (left, gray) and somatosensory condition (right, blue), respectively. Error bars represent standard errors across participants.

Figure 2A shows representative results for the estimated psychometric function prior to and following training in the two conditions (CTL and SOMA). As shown here, the category boundary shifted in the direction of /ε/ following training with somatosensory stimulation (SOMA, solid blue line in the right panel of Figure 2A), indicating that the participants perceived /a/ more than /ε/ as an aftereffect. This shift was not observed following training in the control condition (CTL, solid gray line in the left panel of Figure 2A). Averaged perceptual changes with standard errors are shown in Figure 2B.

The amplitude of the shift was significantly different from zero [−0.163 ± 0.040, average ± s.e., *t* (14) = −4.08, *p* < 0.005] after the training with somatosensory stimulation (SOMA). In the control condition (CTL), the magnitude of the shift was not different than zero [−0.023 ± 0.058, average ± s.e., *t* (14) = −0.41, *p* > 0.6]. A comparison between groups using a LME analysis also showed a significant effect [χ^2^ (1) = 3.88, *p* < 0.05]. These results indicate that the repetitive exposure to somatosensory stimulation during auditory perceptual training can alter the perceptual category boundary as a consequence.

### Perceptual Change During the Training

**FIGURE 3:**
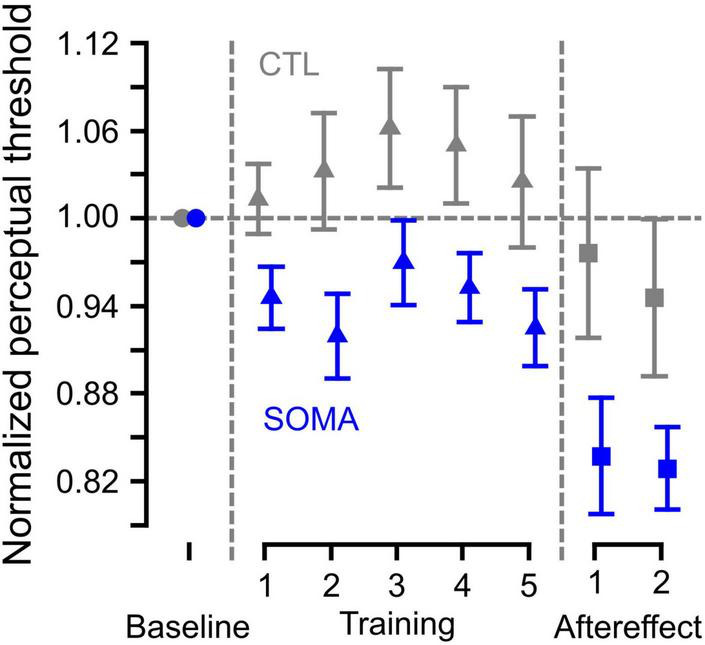
Category boundary values normalized to the baseline category boundary over the course of the experimental procedures. Blue represents the somatosensory condition and gray represents the control condition. Error bars represent standard error across participants.

Figure 3 shows the averaged trajectory of the estimated category boundary over the course of training. In order to examine whether the category boundary changed during the training, we applied the LME analysis to the category boundary estimates obtained over the course of the Training phase. We found that there was no significant interaction between groups (CTL and SOMA) and blocks [χ^2^ (4) = 1.93, *p* > 0.7], indicating that the pattern of change in the category boundary was similar for the two groups. In addition, we did not find a difference across blocks [χ^2^ (4) = 8.70, *p* > 0.06], indicating that there was no change in category boundary over the course of the training. There was a significant overall difference between groups [χ^2^ (1) = 5.19, *p* < 0.03], indicating that the mean value for the category boundary during training in the somatosensory condition was different than that in the control condition. A one-sample *t*-test using the data averaged across blocks showed that values were significantly different from zero in the SOMA condition [*t* (14) = **−**2.83, *p* < 0.02], but not in the CTL condition [*t* (14) = 1.03, *p* > 0.3]. This indicates that participants’ perception in the SOMA condition shifted in the direction of /ε/ during the training phase. This change was not induced in the control condition. The results suggest that there were no temporal changes over the course of training in either group, while somatosensory stimulation induced an overall shift in perception in the experimental condition.

### Persistence of Category Boundary Shift

Although we had limited data to evaluate, we assessed whether the perceptual aftereffect persists following training. We first compared category boundary estimates between the first two (1st set) and the last two blocks (2nd set) of the Aftereffect phase using the LME analysis. There were no significant differences between 1st and 2nd sets [χ^2^ (1) = 0.34, *p* > 0.5]. *Post hoc* tests conducted for the individual conditions found no difference between sets for SOMA (*p* > 0.7) and CTL (*p* > 0.6), respectively. There was a significant interaction between groups and sets [χ^2^ (2) = 11.62, *p* < 0.01]. *Post hoc* tests indicated a significant difference between SOMA and CTL in the 1st set (*p* < 0.05), and a marginal difference in the 2nd set (*p* = 0.073). There is also a significant difference between groups [χ^2^ (1) = 5.14, *p* < 0.05], such that the values for the SOMA group are different than those of the CTL group. Separate one-sample *t*-tests showed that the overall mean in the Aftereffect phase in the SOMA group was reliably different than zero [*t* (14) = **−**5.26, *p* < 0.01], whereas this was not the case for the CTL group [*t* (14) = **−**0.85, *p* > 0.4]. This indicates that the category boundary change following somatosensory stimulation persisted during Aftereffect trials.

**FIGURE 4:**
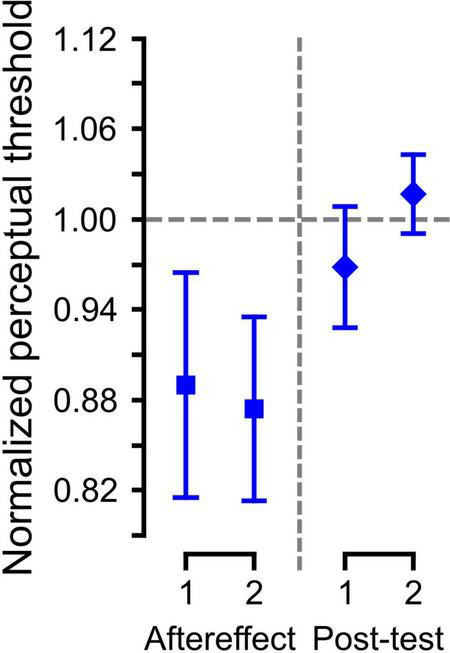
Normalized category boundary in the Aftereffect phase and Post-test (1 week later). Error bars represent the standard error across participants.

We also evaluated if the perceptual change due to paired auditory-somatosensory simulation persisted one-week later. Since only five participants from SOMA group were tested following the one-week delay, we evaluated the effects using five datasets for these participants (one pre-training set, two following training and two after a 1 week delay). The averaged data with standard errors for each set of the Aftereffect and Post-test trials are shown in Figure 4. The LME analysis showed a significant difference between the Aftereffect and Post-test values [χ^2^ (1) = 4.56, *p* < 0.05], but not between the 1st and 2nd sets [χ^2^ (1) = 0.11, *p* > 0.7] nor in the interaction [χ^2^ (2) = 4.95, *p* > 0.08], suggesting that the somatosensory effect was not present one-week later.

## Discussion

The present study examined whether repetitive exposure to somatosensory stimulation in a task which was designed to mirror the pairing of auditory and somatosensory stimulation that occurs during production and speech learning, changes the perceptual representation of speech sounds. We evaluated whether the category boundary between /ε/ and /a/ changed from before to after training with somatosensory stimulation. The somatosensory stimulation involved facial skin deformation in an upward direction. In previous work using a simple perceptual classification task (Ito et al., 2009), this manipulation was found to change the perception of speech sound toward /ε/ when presented with the speech stimuli during training. We found instead that the category boundary between /ε/ and /a/ was in fact shifted toward /ε/, that is, participants perceived /a/ more than /ε/ after training. Although a relatively small number of participants was available for a subsequent post-training test, the shift in the perceptual boundary did not appear to be present 1 week later. The results nevertheless suggest that repetitive exposure to somatosensory inputs associated with facial skin deformation is capable of changing the perceptual representation of speech sounds.

The results of the present study are in line with previous work showing that facial skin deformation changes the perception of speech sounds in on-line testing (Ito et al., 2009; Trudeau-Fisette et al., 2019; Ogane et al., 2020). Repetitive exposure to somatosensory stimulation during speech motor learning may account for the contribution of somatosensation to speech perception (Ohashi and Ito, 2019). The current results are consistent with this hypothesis. Paired auditory-somatosensory input during training, alters subsequent auditory perceptual judgments, suggesting a contribution of somatosensory exposure to speech perception and the presence of a link between speech production and perception.

As mentioned in the Introduction, the category boundary between vowels can be changed when we are repeatedly exposed to one of two vowels, a phenomenon in the speech perception literature known as selective adaptation. Eimas and Corbit (1973) originally showed that the category boundary between /ba/ and /pa/ was shifted toward /ba/, that is, the participants perceived /pa/ more than /ba/ after the training with repetitive exposure of /ba/. The pattern is similar to that of the current finding in which the category boundary shifted toward /ε/ when repetitive somatosensory stimulation, which has been previously shown to modify the perceived speech sound toward /ε/, was applied. A possible mechanism, originally proposed by Eimas and Corbit (1973) is fatigue of a linguistic feature detector as a result of repetitive exposure to the corresponding speech sounds. Kleinschmidt and Jaeger (2016) proposed another possible explanation associated with distributional learning. Although the current results cannot address this debate directly, the current somatosensory effect would fit with either account of selective adaptation. Specifically, in the control condition, we present all values on the speech-sound continuum an equal number of times. As a result, there is no effect on the category boundary, presumably because the entire speech sound representation is affected equally. Both linguistic feature detector and learned distribution accounts would predict a similar result under these conditions. Somatosensory stimulation in the present study serves to modify the perceived sound toward /ε/. Both feature detection fatigue for /ε/ and modification of the stimulation distribution would predict this effect which in turn, may be reflected as a change in the category boundary.

Selective adaptation in speech perception is considered to be an auditory phenomenon when the presented sounds are unambiguous. Previous studies using the McGurk effect (McGurk and MacDonald, 1976) showed that selective adaptation to auditory inputs was induced even when the sound was perceived differently as a result of incongruent visual stimulation (Roberts and Summerfield, 1981; Saldaña and Rosenblum, 1994; Dias et al., 2016). While selective adaptation is observed in visual speech perception (Baart and Vroomen, 2010), Dias et al. (2016) suggested that visual information may not contribute to selective adaptation in the McGurk effect. In the case of the present study, since training with somatosensory stimulation was found to induce a change in the auditory category boundary, the interaction mechanism may be different than in auditory-visual speech perception. This would be consistent with a previous study which found that simultaneous somatosensory and visual stimulation in speech perception did not interact with one other in terms of the behavioral response (Ito et al., 2021). Since somatosensory inputs to speech sounds affect the N1 peak in the auditory ERP (Ito et al., 2014), which is considered to be associated with the initial extraction of vowel related information (Näätänen and Picton, 1987), somatosensory inputs may affect the auditory processing of speech sounds at a lower level of vowel processing. However, somatosensory inputs also affect word segmentation in lexical decisions (Ogane et al., 2020). One future direction is a direct test of the idea that somatosensory stimulation may affect visual speech perception. Baart and Vroomen (2010) showed adaptation in visual speech perception of ambiguous lipread tokens after the exposure to an incongruent sound. Somatosensory stimulation may work in a similar fashion by providing information which disambiguates visual stimuli instead of sounds.

It is important to know how long the training effect lasts. The duration of training phase was limited and as a result, this type of sensory adaptation may not last for a long time. In the case of speech motor learning using altered auditory feedback, the post-training effects on adaptation gradually decrease over the course of the following 100 trials (Purcell and Munhall, 2006; Villacorta et al., 2007). The motion aftereffects described in the Introduction persist for several seconds to minutes. Although it is unknown yet how long selective adaptation lasts, this effect may only persist for a short period, as is the case with sensory adaptation in other modalities. Since the effect of somatosensory training was essentially absent one-week later in the limited number of participants that were tested, the current persistence of a somatosensory aftereffect on speech perception may be similar to other sensory aftereffects. In future investigations, it would be desirable to evaluate shorter periods after training, such as 1 h later, rather than one-week. These types of adaptation including selective adaptation are induced when transient stimulation is presented, and hence when the additional stimulation is removed, particularly after brief periods of training, it is difficult to maintain the adapted perception without receiving additional stimulation. Since this additional stimulation does not exist outside of the laboratory, it may limit the use of the current procedure for speech training or rehabilitation. Nevertheless, the current finding is in line with the more general idea that receiving specific paired of auditory-somatosensory inputs, such as occurs over long periods of time during speech motor training, may underlie a durable contribution of somatosensory inputs to the speech perceptual representation.

In previous work using the same speech sound continuum, in which skin stretch trials were interleaved with no-stretch trials, a change in perception of speech sound toward /ε/ was observed (Ito et al., 2009). In contrast, in the present study, multiple blocks of 100 trials with skin stretch were used. The repetitive pairing of auditory-somatosensory stimulation may have produced a quite different perceptual effect that favored “selective adaptation.” As a result, participants might have perceived /a/ more than /ε/ even in the first 100 trial block.

A potential technical limitation of the present study is that perceptual boundary between speech sounds could not be estimated over a smaller number of trials. While the MCS provides a reasonable estimate of the perceptual boundary, it requires a relatively large number of trials. In the present study, we used 100 trials, and hence, only five estimates of the boundary value were obtained over the course of the current training. The procedure may thus lack the sensitivity needed to correctly capture any changes which might occur. We used a maximum likelihood procedure before and after training as an alternative to improve the possibility of detecting changes over a shorter period of time. However, this method still needs more than ten trials (17 trials in the current case) and requires that participants listen to sounds near to their perceptual boundary rather than over the entire sound continuum, which is the case with the MCS. Due to this technical limitation, it is difficult to characterize perceptual behavior over the course of training. Further investigation is required to better understand the time-course of the current adaptation mechanism.

## Data Availability Statement

The raw data supporting the conclusions of this article will be made available by the authors, without undue reservation.

## Ethics Statement

The studies involving human participants were reviewed and approved by the Comité d’Ethique pour les Recherches non Interventionnelles, Comité d’Ethique pour la Recherche, Grenoble Alpes. The patients/participants provided their written informed consent to participate in this study.

## Author Contributions

TI contributed to the conception and design of the study. TI and RO collected the data, wrote the first draft of the manuscript, involved in subsequent drafts of the manuscript. RO organized the database, performed the statistical analysis (all under TI’s guidance), and produced the figures. Both authors contributed to manuscript revision, read and approved the submitted version.

## Conflict of Interest

The authors declare that the research was conducted in the absence of any commercial or financial relationships that could be construed as a potential conflict of interest.

## Publisher’s Note

All claims expressed in this article are solely those of the authors and do not necessarily represent those of their affiliated organizations, or those of the publisher, the editors and the reviewers. Any product that may be evaluated in this article, or claim that may be made by its manufacturer, is not guaranteed or endorsed by the publisher.
